# The ratio of exogenous Luteinizing hormone to Follicle stimulating hormone administered for controlled ovarian stimulation is associated with oocytes’ number and competence

**DOI:** 10.1042/BSR20190811

**Published:** 2020-01-03

**Authors:** Dragos Albu, Alice Albu

**Affiliations:** 1 Carol Davila University of Medicine and Pharmacy, Faculty of Dentistry, Bucharest, Romania; 2 Reproductive Medicine Department, Medlife Hospital, Bucharest, Romania; 3 Carol Davila University of Medicine and Pharmacy, 2^nd^ Clinical Department, Bucharest, Romania; 4 Endocrinology and Diabetes Department, Elias Hospital, Bucharest, Romania

**Keywords:** follicle stimulating hormone, in vitro fertilization, luteinizing hormone, oocytes’ number

## Abstract

We performed a retrospective study aiming to study the relationship between the ratio of the exogenous luteinizing hormone to follicle stimulating hormone (LH/FSH) administrated for controlled ovarian stimulation (COS) and the number and competence of the oocytes retrieved for *in vitro* fertilization (IVF) or intracytoplasmic sperm injection (ICSI). Eight hundred sixty-eight consecutive infertile patients (mean age 34.54 ± 4.01 years, mean anti-Müllerian hormone (AMH) 2.94 ± 2.07 ng/ml) treated with long agonist protocol and a mixed gonadotropin protocol (human menopausal gonadotropin in association with recombinant FSH (recFSH)) who performed IVF/ICSI between January 2013 and February 2016, were included. Patients with severe male factor were excluded. LH/FSH was calculated based on total doses of the two gonadotropins. We found, after adjustment for confounders, a positive relationship between LH/FSH and the retrieved oocytes’ (β = 0.229, *P*<0.0001) and zygotes’ number (β = 0.144, *P*<0.0001) in the entire study group and in subgroups according to age (<35 and ≥35 years) and ovarian reserve (AMH < 1.1 and ≥ 1.1 ng/ml). The fertilization rate was positively associated with LH/FSH in patients with LH/FSH in the lowest three quartiles (below 0.77) (β = 0.096, *P*=0.034). However, patients in the fourth quartile of LH/FSH had a lower fertilization rate as compared with patients in quartiles 1–3 which, after adjustment for covariates, was only marginally negatively related with LH/FSH (β = −0.108, *P*=0.05). In conclusion, our results suggest that the adequate LH/FSH administrated during COS can improve the oocytes’ and zygotes’ number in IVF/ICSI cycles, but also the fertilization rate when a certain proportion of LH/FSH is not exceeded.

## Introduction

Optimization of the response to assisted reproduction represents a continuous challenge for the practitioners in the reproductive medicine field taking into account the psychological burden of the patients seeking this treatment [1]. The controlled ovarian stimulation (COS) regimen is one of the parameters that can contribute to the success of assisted reproduction [2]. Although the luteinizing hormone (LH) role during the natural cycle is established [3,4], the utility of exogenous LH addition to exogenous follicle stimulating hormone (FSH) for COS is still widely debated. The existing data do not strongly support the universal administration of LH to infertile normogonadotropic patients undergoing COS for *in vitro* fertilization (IVF) [5–7], although some particular categories of patients may benefit. Thus, it was demonstrated that the association of recombinant LH (recLH) with recombinant FSH (recFSH) results in higher ongoing pregnancy rate in comparison with administration of recFSH in women with low serum testosterone levels [8] or of advanced reproductive age (over 35 years old) [9,10]. Similar data are suggested by the studies that compared recFSH with highly purified human menopausal gonadotropin (HP-HMG). HP-HMG is a gonadotropin extracted from the urine of menopausal women which contains both FSH and LH activities, the latter being mainly derived from the urinary hCG present in HP-HMG. These studies found that HP-HMG might be superior to recFSH regarding the live birth rate [11–13], some of the embryo quality parameters and implantation rate of the top-quality embryos [14]. Moreover, it was found a positive relationship between serum HCG level (from HP-HMG composition) and the ongoing pregnancy rate [15,16] and the embryos’ quality [16], supporting the hypothesis that LH activity of HCG is responsible for these outcomes. The most probable explanation for these effects of LH is its influence on the hormonal milieu. Thus, it was demonstrated that both recLH and HMG administration are associated with higher levels of estradiol, androstenedione and testosterone in serum [16] and follicular fluid [16,17], while serum progesterone was higher in patients receiving only recFSH. The two gonadotropins, LH and FSH, have different effects on ovarian steroidogenesis according to the ‘two cells two gonadotropins’ theory [18], therefore, it is expected that various proportions of LH and FSH used in COS to be associated with different levels of ovarian steroids production. In support of this hypothesis, it was shown that various ratios of exogenous LH to FSH are associated with different values of late follicular phase serum progesterone [19] and with different estrogens, androgens and progesterone levels in follicular fluid [20]. However, similar data regarding the relationship between LH to FSH ratio and the full range of the reproductive parameters are lacking. The number of the oocytes retrieved after COS and their competence are significant predictors of the live birth and pregnancy rate [21] and could be influenced by the hormonal ovarian milieu [22]. Still, the possible relationship between the full spectrum of the proportion of doses of exogenous LH and FSH during COS and the number and developmental competence of the oocytes was not previously analyzed. Moreover, studies that reported the oocytes number in patients treated with LH in association with FSH found divergent results, probably due to the heterogeneity of the study protocols and populations [9,23–26]. Therefore, our study aimed to analyze whether there is a relationship between the ratio of doses of LH and FSH administrated for COS in patients performing IVF/intracytoplasmic sperm injection (ICSI) and the number of the oocytes obtained at egg collection and their developmental competence.

## Materials and methods

### Study population

We performed a retrospective study in the Reproductive Medicine Department of Medlife Hospital. The medical data of the patients with all causes of infertility who underwent IVF or ICSI cycles between January 2013 and February 2016 were reviewed. Only patients with all the following data available in the medical records were included: age, cause of infertility, height, weight, doses and type of gonadotropins used for COS, anti-Müllerian hormone (AMH) serum level, the number of oocytes retrieved at eggs collection, the number of zygotes (normally fertilized oocytes the day after IVF/ICSI identified by two pronuclei). Height and weight of the patients were used to calculate the body mass index (BMI) by dividing the weight in kilograms by the square of height in meters. Only patients treated with long agonist protocol in association with a mixed protocol of gonadotropins (HP-HMG in association with recFSH) were selected for the study. The following were considered exclusion criteria: severe male factor as surgically retrieved sperm, cryptozoospermia and non-viable, non-motile sperm after preparation (to avoid the confounding effect of severe male factor on fertilization rate). All the patients gave written informed consent before treatment.

### Stimulation protocols

**Figure 1:**
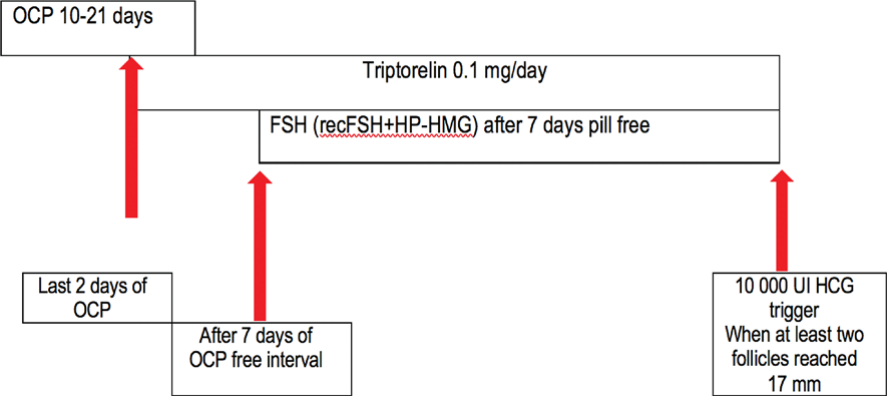
The agonist protocol used for COS in the study group

The common practice in the Reproductive Medicine Department of Medlife Hospital is to administer mixed protocols that include the administration of HMG and recFSH in different proportions in a long agonist protocol, except in a minority of patients which are treated with antagonist protocol for increased risk of ovarian hyperstimulation. Only one clinician established the ovarian stimulation protocol for each case in particular according to age, medical history, BMI, AMH levels, antral follicle count, and, were appropriate, clinician/patients preferences. Dose adjustments were performed according to serum estradiol determinations and ovarian follicles number and dimensions evaluated at transvaginal scans. In all the patients the treatment started with the administration of a combined oral contraceptive pill containing etinilestradiol 0.03 mg and levonorgestrel 0.15 mg for 10–21 days, followed by a long GnRH agonist protocol for pituitary down-regulation (triptorelin 0.1 mg/day starting in the last 2 days of the combined oral contraceptive pill administration till the day before ovulation trigger) (Figure 1). The ovarian stimulation was carried out with recFSH (follitropinum α or follitropinum β) in association with HP-HMG starting after 7 days of pill-free interval. In the antagonist protocol HP-HMG in association with recFSH were administrated starting in the day 2 of a spontaneous menstrual cycle (without previous administration of combined oral contraceptive pills), followed by GnRH antagonist administration (a single dose of cetrotide 0.25 mg in the morning) at day 5 of the cycle. In both protocols oocytes maturation was triggered with a single dose of 10000 UI urinary HCG when at least two follicles reached 17 mm. Oocytes retrieval was performed 36–38 h after HCG administration.

### Study design

A total number of 868 patients were included in the study. The relationship between ratio of the exogenous luteinizing hormone to follicle stimulating hormone (LH/FSH) and retrieved oocytes number, zygotes number and fertilization rate was analyzed in the entire study group and subgroups according to age category (<35 and ≥35 years) and ovarian reserve based on serum AMH level (low ovarian reserve AMH < 1.1 ng/ml and normal ovarian reserve AMH ≥ 1.1 ng/ml) [27]. Patients were considered to be poor responders if they had less than five retrieved oocytes, low responders if the oocytes number was between 5 and 9 and normal responders if the oocytes number was above 9. Because a low number of patients had more than 15 oocytes at eggs collection (*n*=30), they were included in the normal responder category. The causes of infertility were categorized as male factor (less than 5 million spermatozoa with progressive motility), endometriosis (laparoscopically diagnosed), polycystic ovary syndrome (diagnosed by Rotterdam criteria), tubal factor (at least one nonpermeable tube) and idiopathic.

AMH levels were measured by a commercial ELISA kit (AMH Gen II ELISA kit from Beckman-Coulter) with the AMH limit of detection of 0.08 ng/ml. During the study period, the same culture system was used, and the oocytes and zygotes number were reported by the same embryology team. Fertilization rate was calculated by dividing the number of zygotes obtained by the total number of inseminated oocytes for IVF patients and, for ICSI patients, as the total number of zygotes obtained divided by the total number of mature metaphase II injected oocytes. The fertilization rate and zygotes number were used as indicators of oocytes’ developmental competence (the ability of the oocyte to develop into an embryo).

The LH/FSH was calculated based on the total dose of each gonadotropin. One vial of HP-HMG of 75 IU was considered to correspond to 75 IU of human FSH and 75 IU of human LH. The total FSH dose was calculated as the FSH dose from HP-HMG added to the total dose of recFSH.

### Statistical analysis

Statistical analysis was conducted using Statistical Package for the Social Sciences (SPSS) software version 20. A *P*-value below 0.05 was considered indicative of statistical significance. Data are expressed as mean and standard deviation (SD) for continuous variables or percentages for categorical variables, as appropriate. The correlations between the variables analyzed were tested using Pearson product-moment correlation analysis. For comparisons between groups, Student’s *t* test for independent samples was applied for continuous variables. In order to adjust for confounders, we constructed few models of multivariate linear regression (for continuous dependent variables: oocytes number, zygotes number and fertilization rate) and multinomial logistic regression (for nominal dependent variables with more than two values: type of responder). In order to identify nonlinear relationships between LH/FSH and variables studied, the study group was divided into four categories according to LH/FSH quartiles. All the multivariate regression models were adjusted for infertility cause, age, AMH serum level and BMI or other confounding variables (as mentioned were appropriate).

## Results

### Characteristics of the study group

The patients included in the study had a mean age of 34.54 ± 4.01 years and a mean AMH of 2.94 ± 2.07 ng/ml (Table 1). Patients were subdivided in groups according to age: below 35 years (*n*=420 patients, mean age 31.07 ± 2.36 years, mean AMH 3.5 ± 3 ng/ml) and 35 years and older (*n*=448 patients, mean age 37.76 ± 2.34 years, mean AMH 2.4 ± 1.68 ng/ml). They were also divided according to AMH serum level: <1.1 ng/ml (*n*=286 patients) and ≥1.1 ng/ml (*n*=582 patients). The patients were also categorized as poor responders (*n*=232), low responders (*n*=361 patients) and normal responders (*n*=275 patients). Ninety percent of the patients had the LH/FSH ratio between 0.59 and 0.97 (5th and 95th percentiles) and only 10% of the patients had the LH/FSH ratio above 0.85. The cut-off values for the LH/FSH quartiles were: the first quartile below 0.67, second quartile 0.67–0.7, third quartile 0.7–0.77 and fourth quartile above 0.77.

**Table 1:** Clinical and paraclinical characteristics of the study group

	Total	Age < 35 years	Age ≥ 35 years	AMH ≤ 1.1 ng/ml	AMH > 1.1 ng/ml
*Age (years)*	34.54 ± 4.01	31.07 ± 2.36	37.76 ± 2.34	36.19 ± 3.75	33.73 ± 3.94
*AMH (ng/ml)*	2.94 ± 2.07	3.5 ± 3	2.4 ± 1.68	0.6 ± 0.27	3.89 ± 2.18
*BMI (kg/m^2^)*	22.97 ± 2.3	22.84 ± 2.5	22.5 ± 3.94	22.36 ± 3.93	23.6 ± 3.44
*Oocytes (no)*	7.76 ± 4.69	9.09 ± 4.79	6.49 ± 4.25	4.30 ± 3	9.18 ± 4.6
*Embryos (no)*	5.1 ± 3.34	5.78 ± 3.52	4.36 ± 3	3.16 ± 2.35	5.85 ± 3.44
*Fertilization rate*	0.7 ± 0.25	0.69 ± 0.25	0.72 ± 0.26	0.75 ± 0.28	0.69 ± 0.23
*Total FSH dose (IU)*	2444.4 ± 622.7	2324 ± 564.4	2570 ± 634.5	2764.2 ± 716.19	2330.51 ± 540.63
*Total LH dose (IU)*	1746 ± 392	1705 ± 359.1	1793 ± 409.4	1842.36 ± 471.34	1723.35 ± 349.16
*LH/FSH*	0.58 ± 0.13	0.74 ± 0.1	0.71 ± 0.09	0.67 ± 0.08	0.75 ± 0.09

### The relationship between LH/FSH and oocytes number

When the entire study group was analyzed, we found that the number of the oocytes retrieved was positively correlated with LH/FSH (r = 0.383, *P*<0.0001) and AMH serum level (r = 0.498, *P*<0.0001) and negatively with age (r = −0.357, *P*<0.0001) and total doses of LH (r = −0.236, *P*<0.0001) and FSH (r = −0.449, *P*<0.0001).

The positive correlation between LH/FSH and oocytes number was maintained when patients were divided in subgroups according to age (above or below 35 years) and AMH value (below and above 1.1 ng/ml) (Table 2). This positive relationship was maintained in total group and in all subgroups after adjustment for age, AMH serum level, BMI and cause of infertility (Table 2). The negative relationship between oocytes number and total doses of LH and FSH was found in all subgroups of patients after adjustment for confounders (total study group: for LH β = −0.167, *P*<0.0001, for FSH β = −0.307, *P*<0.0001; patients < 35 years: for LH β = −0.222, *P*<0.0001, for FSH β = −0.368, *P*<0.0001; patients ≥ 35 years: for LH β = −0.123, *P*=0.006, for FSH β = −0.255, *P*<0.0001; patients with AMH < 1.1 ng/ml: for LH β = −0.102, *P*=0.05, for FSH β = −0.191, *P*=0.003; patients with AMH ≥ 1.1 ng/ml: for LH β = −0.202, *P*<0.0001, for FSH β = −0.339, *P*<0.0001) (Table 3). In a multinomial regression model we found that patients treated with LH/FSH in the lowest quartiles associated an increased risk of being a poor responder (OR 6.7, *P*<0.0001 for the first quartile and OR 3.1, *P*<0.0001 for the second quartile) or a low responder (OR 5.68, *P*<0.0001 for the first quartile, OR 3.77, *P*<0.0001 for the second quartile and OR 2, *P*=0.003 for the third quartile) in comparison with patients in the forth LH/FSH quartile after adjustment for confounders (Table 4).

**Table 2:** The crude and adjusted correlation between LH/FSH and oocytes and zygotes number

	Oocytes number				Zygotes number			
	r coeff	*P*-value	β coeff*	*P*-value	r coeff	*P*-value	β coeff^†^	*P*-value
*Total*	0.383	<0.0001	0.229	<0.0001	0.284	<0.0001	0.144	<0.0001
*AMH ≤ 1.1 ng/ml*	0.136	0.047	0.138	0.032	0.140	0.041	0.147	0.025
*AMH > 1.1 ng/ml*	0.335	<0.0001	0.230	<0.0001	0.192	<0.0001	0.121	0.008
*Age < 35 years*	0.370	<0.0001	0.247	<0.0001	0.269	<0.0001	0.153	0.004
*Age ≥ 35 years*	0.346	<0.0001	0.220	<0.0001	0.262	<0.0001	0.138	0.007

* β coefficient for LH/FSH in a multivariate linear regression with oocytes number as dependent variable, after adjustment for confounders (age, AMH serum level, BMI and infertility cause).

† β coefficient for LH/FSH in a multivariate linear regression with zygotes number as dependent variable, after adjustment for confounders (age, AMH serum level, BMI and infertility cause).

**Table 3:** The correlation between LH and FSH doses and oocytes number after adjustment for confounders

	Oocytes number			
	FSH dose (IU)		LH dose (IU)	
	β coeff*	*P*-value	β coeff^†^	*P*-value
*Total*	−0.307	<0.0001	−0.167	<0.0001
*AMH < 1.1 ng/ml*	−0.192	0.003	−0.102	0.05
*AMH ≥ 1.1 ng/ml*	−0.339	<0.0001	−0.202	<0.0001
*Age < 35 years*	−0.368	<0.0001	−0.222	<0.0001
*Age ≥ 35 years*	−0.255	<0.0001	−0.123	0.006

* β coefficient for FSH dose in a multivariate regression model with oocytes number as dependent variable, after adjustment for confounders (age, AMH serum level, BMI and infertility cause).

† β coefficient for LH dose in a multivariate regression model with oocytes number as dependent variable, after adjustment for confounders (age, AMH serum level, BMI and infertility cause).

**Table 4:** The risk of being poor and low responder according to the quartile of LH/FSH

	Poor responder		Low responder	
	OR	*P-*value	OR	*P-*value
*First LH/FSH quartile*	6.7	*P*<0.0001	5.68	*P*<0.0001
*Second LH/FSH quartile*	3.1	*P*<0.0001	3.77	*P*<0.0001
*Third LH/FSH quartile*	1.52	NS	2	0.003
*Fourth quartile*	Reference category		Reference category	

### The relationship between LH/FSH and oocytes’ developmental competence parameters

The LH/FSH ratio was positively correlated with the zygotes number (r = 0.284, *P*<0.0001) in the study group and the subgroups of patients according to age and AMH serum level, and this relationship was maintained after adjustment for confounders (Table 2).

**Figure 2:**
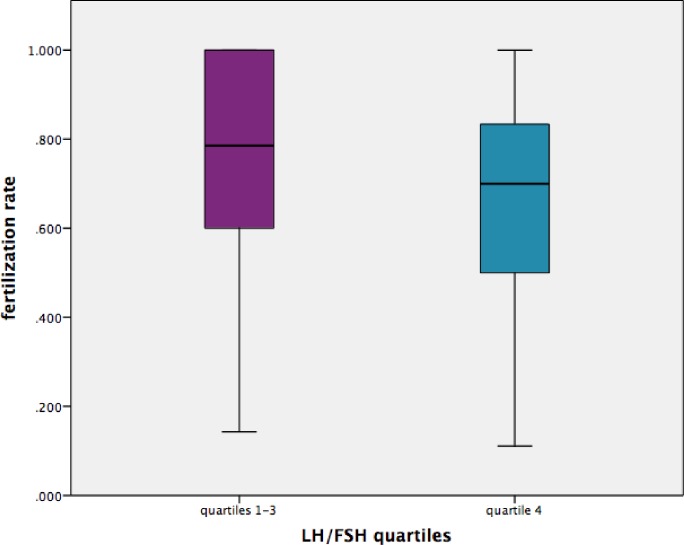
The fertilization rate according to LH/FSH quartile

In the bivariate analysis the fertilization rate was significantly lower in patients in the fourth quartile of LH/FSH in comparison with patients in the other three quartiles (0.67 ± 0.22 versus 0.72 ± 0.26, *P*=0.02) (Figure 2). In a model of multivariate linear regression with fertilization rate as the dependent variable, after adjustment for age, AMH serum level, BMI, infertility cause and oocytes number, the fourth quartile of LH/FSH remained marginally and negatively associated with fertilization rate (β = −0.108, *P*=0.05) (Table 5). When only patients in the first three quartiles of LH/FSH were analyzed, the fertilization rate was weak, but positively correlated with the LH/FSH ratio (β = 0.096, *P*=0.034 after adjustment for confounders) (Table 5). In a multivariate linear regression model with zygotes number as a dependent variable, the oocytes number (β = 0.866, *P*<0.0001) and fertilization rate (β = 0.441, *P*<0.0001), but not LH/FSH, were significant predictors of higher zygotes number.

**Table 5:** The relationship between fertilization rate and LH/FSH

	Fertilization rate (dependent variable)	
	β	*P*-value
Model 1* (fourth LH/FSH quartile)	−0.108^‡^	0.05
Model 2^†^ (LH/FSH as continuous variable)	0.096^§^	0.034

* Model 1: linear multivariate regression model with fertilization rate as dependent variable and LH/FSH quartiles, age, AMH serum level, BMI and infertility cause as independent variables. ^‡^, β for the fourth LH/FH quartile as independent variable in the model 1 of multivariate regression

† Model 2: linear multivariate regression model in patients in the first three quartiles of LH/FSH with fertilization rate as dependent variable and LH/FSH (as continuous variable), age, AMH serum level, BMI, infertility cause and oocytes number as independent variables. ^§^, β for LH/FSH as continuous independent variable in model 2 of multivariate regression.

## Discussion

The results of our study support the importance of an optimal ratio of doses of exogenous LH to FSH used in COS before IVF/ICSI for obtaining a higher oocytes and zygotes number. To the best of our knowledge, this is the first study that reports a linear relationship between LH/FSH doses administrated for COS and oocytes and zygotes number. A possible explanation for the observed positive relationship between gonadotropins ratio and oocytes number can be provided by the differential impact of the two gonadotropins and their doses on the ovarian endocrine milieu. Therefore, in MERIT study it was observed that the administration of HP-HMG, which contains both urinary FSH and LH, is associated with a different hormonal profile in comparison with recFSH administration [16]. Thus, patients who received HP-HMG had higher levels of androstenedione, total testosterone and free androgen index in follicular fluid and serum during ovarian stimulation than those treated with recFSH [16]. Based on the available experimental data, it is believed that androgens act during the early and intermediate stages of folliculogenesis stimulating the transition of the ovarian follicles from the dormant to the growing pool, facilitating the effects of IGF 1 on follicular growth and amplifying the actions of FSH in the FSH-dependent, early antral stages [28]. In the primates’ ovaries, the highest androgen receptor density was found in preantral and antral follicles [29], suggesting the importance of androgens in these stages of development. In humans, a recent study showed a strong association between androgen receptor and FSH receptor mRNA gene expression in antral follicles of 3–13 mm [30], supporting the existence of synergism between androgens and FSH action. Thus, according to these data, androgens are essential in early follicular phase for antral follicle growth and FSH-responsiveness and, therefore, the ovarian microenvironment characterized by higher androgens levels in patients receiving LH can contribute to a higher number of antral follicles available for recruitment under FSH stimulation in COS.

The theoretical basis for the differential effect of the two gonadotropins on the hormonal milieu is provided by the theory of two cells, two gonadotropins. According to this theory, LH stimulates the production of androgens in the theca cells which are then transported to the granulosa cells where they are converted into estrogens under FSH stimulation [18]. Recently it was shown that FSH increases the activity of 3β-HSD, 17β-HSD and aromatase expression in granulosa cells of human ovaries, but does not affect CYP17A1 enzyme expression [31]. Therefore, under exclusive FSH influence ovarian steroidogenesis will be characterized by increased progesterone production and decreased androgen production [31]. When LH is co-administrated with FSH from the beginning of the stimulation, the pregnenolone produced in granulosa cells can be converted by LH action in androgens in theca cells which are abundant in LH/HCG receptors during the early follicular phase [32,33], contributing to an ovarian microenvironment characterized by higher androgens.

The potential benefit of LH addition to FSH for COS was analyzed by several studies which reported divergent results, probably as a consequence of the heterogeneity of the study population and the stimulation protocols. Most of these studies analyzed patients on long agonist protocol and compared COS using only recFSH with patients receiving recFSH in association with recLH [23,24]. These studies found that early LH administration starting on day 1 of stimulation was associated with a higher number of oocytes and embryos [23], while late administration from day 8 or 6 proved no benefit in terms of oocytes, embryos and pregnancy rate [24,25]. On the contrary, studies which analyzed regimens of COS with antagonist protocol found that recLH addition to recFSH was associated with a lower or a similar number of oocytes retrieved in comparison with patients treated only with recFSH [9,26]. A possible explanation for the divergent results of the published studies is the different suppression of the endogenous gonadotropins in agonist versus antagonist protocol. Therefore, in agonist protocol, the suppression of the endogenous LH is assumed to be more profound than in the antagonist protocol, and so the LH substitution could be more important in the former [34]. Moreover, in studies which included patients pretreated with combined oral contraceptives (COCs), endogenous LH could be even more profoundly suppressed [34], making the benefit of LH administration more evident. Moreover, the LH administration from the beginning of stimulation could be essential in order to maximize the benefits on folliculogenesis. In antagonist protocol, the antagonist is administrated after few days of gonadotropin stimulation, allowing early action of endogenous LH before gonadotropins suppression by the antagonist. Therefore, in patients treated with antagonist protocol, it is assumed that early follicular phase LH-driven androgen production is not suppressed, making the addition of LH unnecessary. Moreover, fluctuations of LH level during ovarian stimulation were showed to be detrimental to the reproductive outcome [35], probably offering another explanation for the lack of benefit or even detrimental effect of LH supplementation on oocytes number in antagonist studies.

An important finding of our study is the fact that the positive relationship between LH/FSH and oocytes number was maintained in particular subgroups of patients like those above 35 years old and those with decreased ovarian reserve in which optimization of the ovarian response to stimulation is an essential part of infertility treatment. Moreover, patients treated with the lowest proportions of LH to FSH (in the first and second quartiles, LH/FSH below 0.7) were more likely to be poor responders than normal responders and those treated with LH/FSH ratios in the lowest three quartiles were more likely to be low responders than normal responders. Therefore, our results suggest that we should take into consideration the administration of higher proportions of exogenous LH to FSH ratio to decrease the risk of poor and low response to stimulation.

In our study, a negative correlation between total doses of FSH and LH used for stimulation and oocytes number was observed in the study group and the subgroups of patients according to age and AMH value. One possible explanation is that this negative relationship reflects the higher doses of gonadotropins intuitively administrated in patients with predicted poor response to stimulation. Still, taking into account that the relationship was independent of age and AMH serum level, we can also hypothesis that higher gonadotropin doses can negatively affect the ovarian response to stimulation. Although the data in humans regarding this aspect are scarce, in cattle the FSH doses exceeding a maximal value decreased the number of retrieved and fertilized ova [36,37] and increased the number of degenerated embryos [38], suggesting a negative impact on oocytes quality as well. However, in our patients the doses used for stimulation where rather moderate than excessive, with 90% of the patients receiving mean FSH daily doses below 293 IU and mean LH daily doses below 204 IU (data not showed). Thus, we can hypothesize that even moderately increased doses can negatively impact the oocytes yield. Nevertheless, the positive relationship between LH/FSH ratio and oocytes and embryos number suggests that an optimal proportion between LH and FSH can counteract a possible negative impact of higher gonadotropins doses on ovarian response to stimulation.

We found a marginally negative relationship between fertilization rate and the LH/FSH in the fourth quartile, suggesting that LH/FSH above 0.77 can have a negative impact, although mild, on the fertilization capacity of the oocytes. We also noticed a positive linear relationship between LH/FSH and fertilization rate in patients treated with gonadotropins in the lowest three quartiles of the LH/FSH ratio (below 0.77). Thus, the increasing proportion of LH to FSH seems to be positively related to the fertilization rate until a certain proportion is exceeded. Since the fertilization rate is an indicator of oocytes quality [39], our results suggest that LH/FSH can contribute to oocytes quality. One possible explanation is that the proportion of the two gonadotropins influence the oocytes quality by modulation of the proportion of the androgens and estrogens in the follicular fluid. Thus, it was shown that increasing doses of HCG in association with a fixed dose of FSH administrated for COS are associated with an increase in estradiol, androstenedione and testosterone level in the follicular fluid, but also with a gradually increasing predominance of androgens over estradiol in the follicular fluid [20]. The absolute estrogens levels, but also the estrogens to androgens ratios in the follicular fluid were demonstrated to be positively associated with oocytes quality [40] and with their capacity to give rise to good quality embryos [20]. Experimental *in vitro* studies showed that the non-genomic effect of estradiol on cytoplasmic oocytes maturation could be counteracted by androstenedione, the primary androgen in the follicular fluid [41]. These data in conjunction with our results suggest that increasing ratios of LH/FSH are associated with increasing androgens to estrogens ratios which can negatively impact the oocytes quality when a certain proportion of the two steroids is exceeded. On the other hand, the positive relationship between LH/FSH and fertilization rate at ratios below 0.77 can be the results of the increasing amounts of androgens that serve as a substrate for estrogens production through aromatization without androgen predominance in the follicular milieu. Another mechanism that can be involved in the relationship between LH/FSH and oocytes competence is the regulation of gene expression. Thus, it was shown that recFSH and HMG are associated with different gene expression in granulosa cells of the human ovary [42], HMG administration increasing with more than five-fold the expression of pyruvate kinase, which is the primary source for cellular energy of the oocyte [42]. Therefore, patients treated with increasing amounts of LH in our study and, thus, of HMG could have increasingly competent oocytes with better fertilization rate.

In our patients, LH/FSH was also positively associated with zygotes number, but this association was lost after adjustment for confounders including the oocytes number and fertilization rate which were both independently and positively associated with the zygotes number. These findings suggest that possible mechanism underlying the observed relationship between LH/FSH and zygotes number is its ability to increase the oocytes yield available for fertilization and the fertilization rate, at least in patients in the lower quartiles of LH/FSH. Both the number of oocytes retrieved and of embryos obtained after fertilization were reported as important predictors of IVF success [21,43], being assumed that a high oocytes yield is an indicator of good oocytes quality [43] and oocytes euploidy [44]. In light of these data, the ability of LH/FSH to influence the oocytes and embryos number can offer a way to influence the final IVF outcome.

From the clinical point of view, the correlation between LH/FSH and oocytes’ number and quality, although relatively weak, was statistically significant and deserved to be considered especially in particular situations when the improvement of oocytes and zygotes number is essential. Taking into account that the fourth quartile of LH/FSH is only marginally related to a lower fertilization rate, in clinical practice, when you deal with a patient with a very poor predicted response, seems to be more critical to increase the oocytes number by using higher LH/FSH ratios. However, in situations when you anticipate a low fertilization rate could be a better option to avoid significantly increased LH/FSH.

There are some strengths of our study, including the large sample size and the uniformity of the management of the study group by the same clinician, including the administration of the LH in the form of HCG from HP-HMG in all the patients. Moreover, all the parameters included in the analysis are part of the systematic protocol of the department and were collected in all the patients consecutively treated between January 2013 and February 2016. The main limitation of the study is the relatively narrow range of LH/FSH used in our patients, only 5% of them being treated with LH/FSH below 0.59. Therefore, our findings should be used with caution in patients treated with lower LH/FSH. Another limitation of the study is its retrospective design, which is at least in part overcome by the advantage of observations based on a real-life experience. The HCG-derived LH activity in our patients might have different pharmacological properties [45] and actions [46] from recLH; therefore, it is possible that our results can not be applied to patients treated with the latter product. Moreover, all the patients in our study were treated with a long agonist protocol which has a particular endogenous hormonal profile which probably influences the results of our study. Therefore, our findings should not be extended to other types of protocols.

In conclusion, our study showed that in a long agonist protocol with pre-administration of combined oral contraceptives, the addition of adequate amounts of exogenous LH to exogenous FSH for COS before IVF/ICSI can increase the oocytes and zygotes number, which are significant predictors of IVF success. Our data also suggest a positive association between LH/FSH and fertilization rate when the gonadotropins ratio did not exceed 0.77 and only a marginally negative relationship above this value. Nevertheless, the oocytes number was a strong independent predictor of the zygotes number, suggesting that LH/FSH can contribute to a higher zygotes number by increasing the oocytes yield even in patients with highest LH/FSH in the absence of a severe negative impact on the fertilization rate. However, our findings should be limited to patients treated with long agonist protocol and with HCG-derived LH activity. Due to the complex influence of the LH and FSH on variate reproductive parameters, it remains to be clarified by further studies the impact of LH/FSH on the final IVF outcome.

## Abbreviations

AMH: anti-Müllerian hormone
BMI: body mass index
COS: controlled ovarian stimulation
CYP17A1: cytochrome P450 17A1
FSH: follicle stimulating hormone
GnRH: gonadotropin releasing hormone
hCG: human chorionic gonadotropin
HP-HMG: highly purified human menopausal gonadotropin
ICSI: intracytoplasmic sperm injection
IGF 1: insulin-like growth factor 1
IVF: *in vitro* fertilization
LH: luteinizing hormone
LH/FSH: ratio of the exogenous LH to FSH
MERIT: Menotrophin versus Recombinant FSH *in vitro* Fertilization
OR: odd ratio
recFSH: recombinant FSH
recLH: recombinant LH
SD: standard deviation
SPSS: Statistical Package for the Social Sciences
3β-HSD: 3 beta hydroxysteroid dehydrogenase
17β-HSD: 17 beta hydroxysteroid dehydrogenase

## References

[1] Massarotti C., Gentile G., Ferreccio C., Scaruffi P., Remorgida V. and Anserini P. (2019) Impact of infertility and infertility treatments on quality of life and levels of anxiety and depression in women undergoing *in vitro* fertilization. Gynecol. Endocrinol. 35, 485–489 30612477 10.1080/09513590.2018.1540575

[2] Bosch E., Labarta E., Kolibianakis E., Rosen M. and Meldrum D. (2016) Regimen of ovarian stimulation affects oocyte and therefore embryo quality. Fertil. Steril. 105, 560–570 10.1016/j.fertnstert.2016.01.022 26826273

[3] Balasch J., Miro F., Burzaco I. et al. (1995) The role of luteinizing hormone in human follicle development and oocyte fertility: evidence from in-vitro fertilization in a woman with long-standing hypogonadotrophic hypogonadism and using recombinant human follicle stimulating hormone. Hum. Reprod. 10, 1678–1683 10.1093/oxfordjournals.humrep.a136154 8582960

[4] The European Recombinant Human LH Study Group (1998) Recombinant human luteinizing hormone (LH) to support recombinant human follicle-stimulating hormone (FSH)-induced follicular development in LH- and FSH-deficient anovulatory women: a dose-finding study. J. Clin. Endocrinol. Metab. 83, 1507–1514 9589647 10.1210/jcem.83.5.4770

[5] Mochtar M.H., Van der Veen F., Ziech M. and van Wely M. (2007) Recombinant Luteinizing Hormone (rLH) for controlled ovarian hyperstimulation in assisted reproductive cycles. Cochrane Database Syst. Rev. CD005070 10.1002/14651858.CD005070.pub2 17443569

[6] Mochtar M.H., Danhof N.A., Ayeleke R.O., Van der Veen F. and van Wely M. (2017) Recombinant luteinizing hormone (rLH) and recombinant follicle stimulating hormone (rFSH) for ovarian stimulation in IVF/ICSI cycles. Cochrane Database Syst. Rev. 5, CD005070 28537052 10.1002/14651858.CD005070.pub3 PMC6481753

[7] Kolibianakis E.M., Collins J., Tarlatzis B.C., Devroey P., Diedrich K. and Griesinger G. (2006) Among patients treated for IVF with gonadotrophins and GnRH analogues, is the probability of live birth dependent on the type of analogue used? A systematic review and meta-analysis Hum. Reprod. Update 12, 651–671 10.1093/humupd/dml038 16920869

[8] Bosch E., Labarta E., Vidal C. et al. (2011) The relationship between serum androgen levels and the need of LH administration during controlled ovarian stimulation for *in vitro* fertilization: an explorative study. Hum. Reprod. 26, i26 10.1093/humrep/26.s1.18

[9] Bosch E., Labarta E., Crespo J., Simon C., Remohi J. and Pellicer A. (2011) Impact of luteinizing hormone administration on gonadotropin-releasing hormone antagonist cycles: an age-adjusted analysis. Fertil. Steril. 95, 1031–1036 10.1016/j.fertnstert.2010.10.021 21067717

[10] Hill M.J., Levens E.D., Levy G. et al. (2012) The use of recombinant luteinizing hormone in patients undergoing assisted reproductive techniques with advanced reproductive age: a systematic review and meta-analysis. Fertil. Steril. 97, 1108.e1–1114.e1 10.1016/j.fertnstert.2012.01.130 22365075

[11] Al-Inany H.G., Abou-Setta A.M., Aboulghar M.A., Mansour R.T. and Serour G.I. (2008) Efficacy and safety of human menopausal gonadotrophins versus recombinant FSH: a meta-analysis. Reprod. Biomed. Online 16, 81–88 10.1016/S1472-6483(10)60559-7 18252052

[12] Platteau P., Nyboe Andersen A., Loft A., Smitz J., Danglas P. and Devroey P. (2008) Highly purified HMG versus recombinant FSH for ovarian stimulation in IVF cycles. Reprod. Biomed. Online 17, 190–198 10.1016/S1472-6483(10)60194-0 18681992

[13] Coomarasamy A., Afnan M., Cheema D., van der Veen F., Bossuyt P.M. and van Wely M. (2008) Urinary hMG versus recombinant FSH for controlled ovarian hyperstimulation following an agonist long down-regulation protocol in IVF or ICSI treatment: a systematic review and meta-analysis. Hum. Reprod. 23, 310–315 10.1093/humrep/dem305 18056719

[14] Ziebe S., Lundin K., Janssens R., Helmgaard L., Arce J.C. and MERIT (Menotrophin vs Recombinant FSH in vitro Fertilisation Trial) Group (2007) Influence of ovarian stimulation with HP-hMG or recombinant FSH on embryo quality parameters in patients undergoing IVF. Hum. Reprod. 22, 2404–2413 17640944 10.1093/humrep/dem221

[15] Platteau P., Smitz J., Albano C., Sørensen P., Arce J.C. and Devroey P. (2004) Exogenous luteinizing hormone activity may influence the treatment outcome in in vitro fertilization but not in intracytoplasmic sperm injection cycles. Fertil. Steril. 81, 1401–1404 10.1016/j.fertnstert.2003.09.077 15136112

[16] Smitz J., Andersen A.N., Devroey P. and Arce JC for the MERIT* Group Endocrine profile in serum and follicular fluid differs after ovarian stimulation with HP-hMG or recombinant FSH in IVF patients. Hum Reprod. 22, 2007–687 10.1093/humrep/del445 17110397

[17] Bosch E., Pau E., Albert C., Zuzuarregui J.L., Remohí J. and Pellicer A. (2006) Impact of different amounts of LH in controlled ovarian hyperstimulation oocyte donation cycles. Fertil. Steril. 86, S425 10.1016/j.fertnstert.2006.07.1173

[18] Hillier S.G., Whitelaw P.F. and Smyth C.D. (1994) Follicular oestrogen synthesis: the ‘two-cell, two-gonadotrophin’ model revisited. Mol. Cell. Endocrinol. 100, 51–54 10.1016/0303-7207(94)90278-X 8056158

[19] Werner M.D., Forman E.J., Hong K.H., Franasiak J.M., Molinaro T.A. and Scott R.T. Jr (2014) Defining the “sweet spot” for administered luteinizing hormone-to-follicle-stimulating hormone gonadotropin ratios during ovarian stimulation to protect against a clinically significant late follicular increase in progesterone: an analysis of 10,280 first in vitro fertilization cycles. Fertil. Steril. 102, 1312–1317 10.1016/j.fertnstert.2014.07.766 25150393

[20] Thuesen L.L., Andersen A.N., Loft A. and Smitz J. (2014) Intrafollicular endocrine milieu after addition of hCG to recombinant FSH during controlled ovarian stimulation for *in vitro* fertilization. J. Clin. Endocrinol. Metab. 99, 517–526 10.1210/jc.2013-1528 24297796

[21] Hariton E., Kim K., Mumford S.L. et al. (2017) Total number of oocytes and zygotes are predictive of live births pregnancy in fresh donor oocytes IVF cycles. Fertil. Steril. 108, 262–268 10.1016/j.fertnstert.2017.05.021 28601410 PMC5545054

[22] Dumesic D.A., Meldrum D.R., Katz-Jaffe M.G., Krisher R.L. and Schoolcraft W.B. (2015) Oocyte environment: follicular fluid and cumulus cells are critical for oocyte health. Fertil. Steril. 103, 303–316 10.1016/j.fertnstert.2014.11.015 25497448

[23] Franco J.G., Baruffi R.L.R., Oliveira J.B.A. et al. (2009) Effects of recombinant LH supplementation to recombinant FSH during induced ovarian stimulation in the GnRH-agonist protocol: a matched case-control study. Reprod. Biol. Endocrinol. 7, 58 10.1186/1477-7827-7-58 19497101 PMC2701434

[24] Humaidan P., Bungum M., Bungum L. and Andersen C.Y. (2004) Effects of recombinant LH supplementation in women undergoing assisted reproduction with GnRH agonist down-regulation and stimulation with recombinant FSH: an opening study. Reprod. Biomed. Online 8, 635–643 10.1016/S1472-6483(10)61643-4 15169576

[25] Marrs R., Meldrum D., Muasher S., Schoolcraft W., Werlin L. and Kelly E. (2003) Randomized trial to compare the effect of recombinant human FSH (follitropin alfa) with or without recombinant human LH in women undergoing assisted reproduction treatment. Reprod. Biomed. Online 8, 175–182 10.1016/S1472-6483(10)60513-5 14989794

[26] König T.E., van der Houwen L.E.E., Overbeek A. et al. (2013) Recombinant LH supplementation to a standard GnRH antagonist protocol in women of 35 years or older undergoing IVF/ICSI: a randomized controlled multicentre study. Hum. Reprod. 28, 2804–2812 10.1093/humrep/det266 23838159

[27] Ferraretti A.P., La Marca A., Fauser B.C., Tarlatzis B. and Nargund G. (2011) Gianarolion L on behalf of the ESHRE working group on Poor Ovarian Response Definition: ESHRE consensus on the definition of ‘poor response’ to ovarian stimulation for *in vitro* fertilization: the Bologna criteria. Hum. Reprod. 26, 1616–1624 10.1093/humrep/der092 21505041

[28] Gervásio C.G., Bernuci M.P., Silva-de-Sá M.F. and Rosa-E-Silva A.C. (2014) The role of androgen hormones in early follicular development. ISRN Obstet. Gynecol. 2014, 818010 25006485 10.1155/2014/818010 PMC4003798

[29] Weil S.J., Vendola K., Zhou J. et al. (1998) Androgen receptor gene expression in the primate ovary: cellular localization, regulation, and functional correlations. J. Clin. Endocrinol. Metab. 83, 2479–2485 10.1210/jcem.83.7.4917 9661631

[30] Kristensen S.G., Mamsen L.S., Jeppesen J.V. et al. (2017) Hallmarks of human small antral follicle development: implications for regulation of ovarian steroidogenesis and selection of the dominant follicle. Front. Endocrinol. (Lausanne) 8, 376 10.3389/fendo.2017.00376 29375481 PMC5770355

[31] Oktem O., Akin N., Bildik G. et al. (2017) FSH Stimulation promotes progesterone synthesis and output from human granulosa cells without luteinization. Hum. Reprod. 32, 643–652 10.1093/humrep/dex010 28158500

[32] Zeleznik A.J., Midgley A.R. and Reichert L.E. (1974) Granulosa cell maturation in the rat: increased binding of human chorionic gonadotropin following treatment with follicle-stimulating hormone *in vivo*. Endocrinology 95, 818–825 10.1210/endo-95-3-818 4368756

[33] Hillier S.G. (2001) Gonadotropic control of ovarian follicular growth and development. Mol. Cell. Endocrinol. 179, 39–46 10.1016/S0303-7207(01)00469-5 11420129

[34] Huirne J.A., Homburg R. and Lambalk C.B. (2007) Are GnRH antagonists comparable to agonists for use in IVF? Hum. Reprod. 22, 2805–2813 10.1093/humrep/dem270 17872909

[35] Huirne J.A.F., van Loenen A.C.D., Schats R. et al. (2005) Dose-finding study of daily GnRH antagonist for the prevention of premature LH surges in IVF/ICSI patients: optimal changes in LH and progesterone for clinical pregnancy. Hum. Reprod. 20, 359–367 10.1093/humrep/deh601 15567880

[36] McGowan M.R., Braithwaite M., Jochle W. and Maplecoft R.J. (1985) Superovulation of beef heifers with Pergonal (HMG): a dose response trial. Theriogenology 24, 173–184 10.1016/0093-691X(85)90181-5 16726070

[37] Souza A.H., Sartori R., Guenther J.N., Caraviello D., Monson R. and Wiltbank M. (2007) Effect of semen source and dose of FSH on superovulatory response and embryo production in Holstein heifers. Anim. Reprod. Sci. 4, 70–76

[38] Wilson J.M., Jones A.L., Moore K., Looney C.R. and Bondioli K.R. (1993) Superovulation of cattle with a recombinant-DNA bovine follicle stimulating hormone. Anim. Reprod. Sci. 33, 71–82 10.1016/0378-4320(93)90107-3

[39] Albertini D.F., Sanfins A. and Combelles C.M. (2003) Origins and manifestations of oocyte maturation competencies. Reprod. Biomed. Online 6, 410–415 10.1016/S1472-6483(10)62159-1 12831584

[40] Xia P. and Younglai E.V. (2000) Relationship between steroid concentrations in ovarian follicular fluid and oocyte morphology in patients undergoing intracytoplasmic sperm injection (ICSI) treatment. J. Reprod. Fertil. 118, 229–233 10.1530/jrf.0.1180229 10864786

[41] Tesarik J. and Mendoza C. (1997) Direct non-genomic effects of follicular steroids on maturing human oocytes: oestrogen versus androgen antagonism. Hum. Reprod. Update 3, 95–100 10.1093/humupd/3.2.95 9286733

[42] Brannian J., Eyster K., Mueller B.A., Bietz M.G. and Hansen K. (2010) Differential gene expression in human granulosa cells from recombinant FSH versus human menopausal gonadotropin ovarian stimulation protocols. Reprod. Biol. Endocrinol. 8, 25 10.1186/1477-7827-8-25 20226040 PMC2842272

[43] Sunkara S.K., Khalaf Y., Maheshwari A., Seed P. and Coomarasamy A. (2014) Association between response to ovarian stimulation and miscarriage following IVF: an analysis of 124 351 IVF pregnancies. Hum. Reprod. 29, 1218–1224 10.1093/humrep/deu053 24651128

[44] Venetis C.A., Tilia L., Panlilio E. and Kan A. (2019) Is more better? A higher oocyte yield is independently associated with more day-3 euploid embryos after ICSI Hum. Reprod. 34, 79–83 10.1093/humrep/dey342 30476100

[45] Choi J. and Smitz J. (2014) Luteinizing hormone and human chorionic gonadotropin: origins of difference. Mol. Cell. Endocrinol. 383, 203–213 10.1016/j.mce.2013.12.009 24365330

[46] Grøndahl M.L., Borup R., Lee Y.B., Myrhøj V., Meinertz H. and Sørensen S. (2009) Differences in gene expression of granulosa cells from women undergoing controlled ovarian hyperstimulation with either recombinant follicle-stimulating hormone or highly purified human menopausal gonadotropin. Fertil. Steril. 91, 1820–1830 10.1016/j.fertnstert.2008.02.137 18439596

